# Supplementary material for: The accuracy of three-dimensional facial scan obtained from three different 3d scanners
Source: PLoS One. 2025 May 23;20(5):e0322358. doi: 10.1371/journal.pone.0322358 (PMC12101780; doi:10.1371/journal.pone.0322358)
Supplement: S2 Table — T: Trueness, P: Precision, PMC: Planmeca Proface, Ein: EnScanH2, APP: EM3D scanner application, Rcheek: Right cheek unit, Lcheek: Left cheek unit, Nose: nose unit, Perioral: Perioral unit, Chin: Mental unit. (PDF) [file pone.0322358.s002.pdf]

Descriptives

			Statistic	Std. Error
T_Face_PMC	Mean		.6520	.04903
	95% Confidence Interval for Mean	Lower Bound	.5411	
		Upper Bound	.7629	
	5% Trimmed Mean		.6417	
	Median		.6050	
	Variance		.024	
	Std. Deviation		.15505	
	Minimum		.50	
	Maximum		.99	
	Range		.49	
	Interquartile Range		.18	
	Skewness		1.444	.687
	Kurtosis		1.549	1.334
P_Face_PMC	Mean		.6260	.04576
	95% Confidence Interval for Mean	Lower Bound	.5225	
		Upper Bound	.7295	
	5% Trimmed Mean		.6161	
	Median		.5800	
	Variance		.021	
	Std. Deviation		.14470	
	Minimum		.49	
	Maximum		.94	
	Range		.45	
	Interquartile Range		.13	
	Skewness		1.571	.687
	Kurtosis		1.662	1.334
T_Face_Ein	Mean		.7150	.09013
	95% Confidence Interval for Mean	Lower Bound	.5111	
		Upper Bound	.9189	
	5% Trimmed Mean		.7161	
	Median		.7650	
	Variance		.081	
	Std. Deviation		.28500	
	Minimum		.27	
	Maximum		1.14	
	Range		.87	
	Interquartile Range		.45	
	Skewness		-.382	.687
	Kurtosis		-.555	1.334
P_Face_Ein	Mean		.6860	.08689
	95% Confidence Interval for Mean	Lower Bound	.4894	
		Upper Bound	.8826	
	5% Trimmed Mean		.6844	
	Median		.7150	
	Variance		.075	
	Std. Deviation		.27476	
	Minimum		.27	
	Maximum		1.13	
	Range		.86	
	Interquartile Range		.43	
	Skewness		-.222	.687
	Kurtosis		-.419	1.334
T_Face_App	Mean		.6190	.03808
	95% Confidence Interval for Mean	Lower Bound	.5329	
		Upper Bound	.7051	
	5% Trimmed Mean		.6228	
	Median		.6850	
	Variance		.014	
	Std. Deviation		.12041	
	Minimum		.43	
	Maximum		.74	
	Range		.31	
	Interquartile Range		.24	
	Skewness		-.763	.687
	Kurtosis		-1.370	1.334
P_Face_App	Mean		.5960	.03566
	95% Confidence Interval for Mean	Lower Bound	.5153	
		Upper Bound	.6767	
	5% Trimmed Mean		.5994	
	Median		.6450	
	Variance		.013	
	Std. Deviation		.11276	
	Minimum		.43	
	Maximum		.70	
	Range		.27	
	Interquartile Range		.24	
	Skewness		-.745	.687
	Kurtosis		-1.415	1.334

T_Rcheek_PMC	Mean		.6360	.06233
	95% Confidence Interval for Mean	Lower Bound	.4950	
		Upper Bound	.7770	
	5% Trimmed Mean		.6222	
	Median		.6100	
	Variance		.039	
	Std. Deviation		.19710	
	Minimum		.44	
	Maximum		1.08	
	Range		.64	
	Interquartile Range		.29	
	Skewness		1.308	.687
	Kurtosis		1.940	1.334
P_Rcheek_PMC	Mean		.5720	.04661
	95% Confidence Interval for Mean	Lower Bound	.4666	
		Upper Bound	.6774	
	5% Trimmed Mean		.5600	
	Median		.5400	
	Variance		.022	
	Std. Deviation		.14741	
	Minimum		.43	
	Maximum		.93	
	Range		.50	
	Interquartile Range		.16	
	Skewness		1.788	.687
	Kurtosis		3.712	1.334
T_Rcheek_Ein	Mean		.7320	.09224
	95% Confidence Interval for Mean	Lower Bound	.5233	
		Upper Bound	.9407	
	5% Trimmed Mean		.7389	
	Median		.8500	
	Variance		.085	
	Std. Deviation		.29169	
	Minimum		.28	
	Maximum		1.06	
	Range		.78	
	Interquartile Range		.56	
	Skewness		-.659	.687
	Kurtosis		-1.291	1.334
P_Rcheek_Ein	Mean		.6040	.06672
	95% Confidence Interval for Mean	Lower Bound	.4531	
		Upper Bound	.7549	
	5% Trimmed Mean		.6067	
	Median		.6850	
	Variance		.045	
	Std. Deviation		.21099	
	Minimum		.26	
	Maximum		.90	
	Range		.64	
	Interquartile Range		.34	
	Skewness		-.574	.687
	Kurtosis		-.754	1.334
T_Rcheek_App	Mean		.6170	.05097
	95% Confidence Interval for Mean	Lower Bound	.5017	
		Upper Bound	.7323	
	5% Trimmed Mean		.6156	
	Median		.5750	
	Variance		.026	
	Std. Deviation		.16118	
	Minimum		.40	
	Maximum		.86	
	Range		.46	
	Interquartile Range		.28	
	Skewness		.166	.687
	Kurtosis		-1.397	1.334
P_Rcheek_App	Mean		.5530	.04284
	95% Confidence Interval for Mean	Lower Bound	.4561	
		Upper Bound	.6499	
	5% Trimmed Mean		.5528	
	Median		.5350	
	Variance		.018	
	Std. Deviation		.13549	
	Minimum		.36	
	Maximum		.75	
	Range		.39	
	Interquartile Range		.27	
	Skewness		.308	.687
	Kurtosis		-1.126	1.334

T_Lcheek_PMC	Mean		.7940	.09057
	95% Confidence Interval for Mean	Lower Bound	.5891	
		Upper Bound	.9989	
	5% Trimmed Mean		.7783	
	Median		.7500	
	Variance		.082	
	Std. Deviation		.28640	
	Minimum		.53	
	Maximum		1.34	
	Range		.81	
	Interquartile Range		.41	
	Skewness		1.141	.687
	Kurtosis		.308	1.334
P_Lcheek_PMC	Mean		.7080	.08656
	95% Confidence Interval for Mean	Lower Bound	.5122	
		Upper Bound	.9038	
	5% Trimmed Mean		.6983	
	Median		.6150	
	Variance		.075	
	Std. Deviation		.27373	
	Minimum		.37	
	Maximum		1.22	
	Range		.85	
	Interquartile Range		.38	
	Skewness		1.011	.687
	Kurtosis		.100	1.334
T_Lcheek_Ein	Mean		.7990	.13544
	95% Confidence Interval for Mean	Lower Bound	.4926	
		Upper Bound	1.1054	
	5% Trimmed Mean		.7994	
	Median		.8150	
	Variance		.183	
	Std. Deviation		.42829	
	Minimum		.18	
	Maximum		1.41	
	Range		1.23	
	Interquartile Range		.84	
	Skewness		.017	.687
	Kurtosis		-1.305	1.334
P_Lcheek_Ein	Mean		.5950	.08916
	95% Confidence Interval for Mean	Lower Bound	.3933	
		Upper Bound	.7967	
	5% Trimmed Mean		.5906	
	Median		.6550	
	Variance		.079	
	Std. Deviation		.28195	
	Minimum		.18	
	Maximum		1.09	
	Range		.91	
	Interquartile Range		.39	
	Skewness		.074	.687
	Kurtosis		-.294	1.334
T_Lcheek_App	Mean		.6120	.06162
	95% Confidence Interval for Mean	Lower Bound	.4726	
		Upper Bound	.7514	
	5% Trimmed Mean		.6167	
	Median		.6700	
	Variance		.038	
	Std. Deviation		.19487	
	Minimum		.31	
	Maximum		.83	
	Range		.52	
	Interquartile Range		.39	
	Skewness		-.530	.687
	Kurtosis		-1.228	1.334
P_Lcheek_App	Mean		.5520	.05587
	95% Confidence Interval for Mean	Lower Bound	.4256	
		Upper Bound	.6784	
	5% Trimmed Mean		.5561	
	Median		.6250	
	Variance		.031	
	Std. Deviation		.17669	
	Minimum		.27	
	Maximum		.76	
	Range		.49	
	Interquartile Range		.30	
	Skewness		-.471	.687
	Kurtosis		-1.549	1.334

T_Nose_PMC	Mean		.3780	.02279
	95% Confidence Interval for Mean	Lower Bound	.3264	
		Upper Bound	.4296	
	5% Trimmed Mean		.3744	
	Median		.3750	
	Variance		.005	
	Std. Deviation		.07208	
	Minimum		.29	
	Maximum		.53	
	Range		.24	
	Interquartile Range		.10	
	Skewness		.949	.687
	Kurtosis		.881	1.334
P_Nose_PMC	Mean		.2930	.01146
	95% Confidence Interval for Mean	Lower Bound	.2671	
		Upper Bound	.3189	
	5% Trimmed Mean		.2911	
	Median		.2900	
	Variance		.001	
	Std. Deviation		.03622	
	Minimum		.25	
	Maximum		.37	
	Range		.12	
	Interquartile Range		.05	
	Skewness		1.030	.687
	Kurtosis		1.096	1.334
T_Nose_Ein	Mean		.4840	.06587
	95% Confidence Interval for Mean	Lower Bound	.3350	
		Upper Bound	.6330	
	5% Trimmed Mean		.4750	
	Median		.4400	
	Variance		.043	
	Std. Deviation		.20828	
	Minimum		.27	
	Maximum		.86	
	Range		.59	
	Interquartile Range		.36	
	Skewness		.807	.687
	Kurtosis		-.264	1.334
P_Nose_Ein	Mean		.4630	.06722
	95% Confidence Interval for Mean	Lower Bound	.3109	
		Upper Bound	.6151	
	5% Trimmed Mean		.4544	
	Median		.4200	
	Variance		.045	
	Std. Deviation		.21255	
	Minimum		.24	
	Maximum		.84	
	Range		.60	
	Interquartile Range		.35	
	Skewness		.851	.687
	Kurtosis		-.264	1.334
T_Nose_App	Mean		.5160	.05980
	95% Confidence Interval for Mean	Lower Bound	.3807	
		Upper Bound	.6513	
	5% Trimmed Mean		.5067	
	Median		.4800	
	Variance		.036	
	Std. Deviation		.18910	
	Minimum		.28	
	Maximum		.92	
	Range		.64	
	Interquartile Range		.21	
	Skewness		1.181	.687
	Kurtosis		1.315	1.334
P_Nose_App	Mean		.4620	.06092
	95% Confidence Interval for Mean	Lower Bound	.3242	
		Upper Bound	.5998	
	5% Trimmed Mean		.4522	
	Median		.3750	
	Variance		.037	
	Std. Deviation		.19263	
	Minimum		.25	
	Maximum		.85	
	Range		.60	
	Interquartile Range		.25	
	Skewness		1.189	.687
	Kurtosis		.537	1.334

T_Perioral_PMC	Mean		.5040	.05342
	95% Confidence Interval for Mean	Lower Bound	.3832	
		Upper Bound	.6248	
	5% Trimmed Mean		.5017	
	Median		.4850	
	Variance		.029	
	Std. Deviation		.16893	
	Minimum		.24	
	Maximum		.81	
	Range		.57	
	Interquartile Range		.25	
	Skewness		.381	.687
	Kurtosis		-.092	1.334
P_Perioral_PMC	Mean		.4470	.05369
	95% Confidence Interval for Mean	Lower Bound	.3256	
		Upper Bound	.5684	
	5% Trimmed Mean		.4433	
	Median		.4250	
	Variance		.029	
	Std. Deviation		.16977	
	Minimum		.23	
	Maximum		.73	
	Range		.50	
	Interquartile Range		.28	
	Skewness		.271	.687
	Kurtosis		-.972	1.334
T_Perioral_Ein	Mean		.7400	.10726
	95% Confidence Interval for Mean	Lower Bound	.4974	
		Upper Bound	.9826	
	5% Trimmed Mean		.7322	
	Median		.7000	
	Variance		.115	
	Std. Deviation		.33918	
	Minimum		.32	
	Maximum		1.30	
	Range		.98	
	Interquartile Range		.58	
	Skewness		.539	.687
	Kurtosis		-.639	1.334
P_Perioral_Ein	Mean		.6410	.08781
	95% Confidence Interval for Mean	Lower Bound	.4424	
		Upper Bound	.8396	
	5% Trimmed Mean		.6272	
	Median		.6050	
	Variance		.077	
	Std. Deviation		.27767	
	Minimum		.29	
	Maximum		1.24	
	Range		.95	
	Interquartile Range		.33	
	Skewness		1.048	.687
	Kurtosis		1.498	1.334
T_Perioral_App	Mean		.6520	.07121
	95% Confidence Interval for Mean	Lower Bound	.4909	
		Upper Bound	.8131	
	5% Trimmed Mean		.6456	
	Median		.6700	
	Variance		.051	
	Std. Deviation		.22518	
	Minimum		.39	
	Maximum		1.03	
	Range		.64	
	Interquartile Range		.34	
	Skewness		.589	.687
	Kurtosis		-.594	1.334
P_Perioral_App	Mean		.6120	.06576
	95% Confidence Interval for Mean	Lower Bound	.4632	
		Upper Bound	.7608	
	5% Trimmed Mean		.6022	
	Median		.6200	
	Variance		.043	
	Std. Deviation		.20794	
	Minimum		.37	
	Maximum		1.03	
	Range		.66	
	Interquartile Range		.32	
	Skewness		.742	.687
	Kurtosis		.338	1.334

T_Chin_PMC	Mean		.4920	.04407
	95% Confidence Interval for Mean	Lower Bound	.3923	
		Upper Bound	.5917	
	5% Trimmed Mean		.4911	
	Median		.4550	
	Variance		.019	
	Std. Deviation		.13935	
	Minimum		.28	
	Maximum		.72	
	Range		.44	
	Interquartile Range		.21	
	Skewness		.355	.687
	Kurtosis		-.678	1.334
P_Chin_PMC	Mean		.4460	.04188
	95% Confidence Interval for Mean	Lower Bound	.3513	
		Upper Bound	.5407	
	5% Trimmed Mean		.4433	
	Median		.4450	
	Variance		.018	
	Std. Deviation		.13243	
	Minimum		.27	
	Maximum		.67	
	Range		.40	
	Interquartile Range		.21	
	Skewness		.295	.687
	Kurtosis		-.722	1.334
T_Chin_Ein	Mean		.5390	.07801
	95% Confidence Interval for Mean	Lower Bound	.3625	
		Upper Bound	.7155	
	5% Trimmed Mean		.5311	
	Median		.4300	
	Variance		.061	
	Std. Deviation		.24669	
	Minimum		.26	
	Maximum		.96	
	Range		.70	
	Interquartile Range		.43	
	Skewness		.729	.687
	Kurtosis		-.908	1.334
P_Chin_Ein	Mean		.4460	.06938
	95% Confidence Interval for Mean	Lower Bound	.2890	
		Upper Bound	.6030	
	5% Trimmed Mean		.4378	
	Median		.4100	
	Variance		.048	
	Std. Deviation		.21940	
	Minimum		.21	
	Maximum		.83	
	Range		.62	
	Interquartile Range		.31	
	Skewness		1.025	.687
	Kurtosis		.189	1.334
T_Chin_App	Mean		.6060	.04766
	95% Confidence Interval for Mean	Lower Bound	.4982	
		Upper Bound	.7138	
	5% Trimmed Mean		.6039	
	Median		.5850	
	Variance		.023	
	Std. Deviation		.15072	
	Minimum		.34	
	Maximum		.91	
	Range		.57	
	Interquartile Range		.17	
	Skewness		.422	.687
	Kurtosis		1.571	1.334
P_Chin_App	Mean		.5290	.05152
	95% Confidence Interval for Mean	Lower Bound	.4125	
		Upper Bound	.6455	
	5% Trimmed Mean		.5250	
	Median		.5000	
	Variance		.027	
	Std. Deviation		.16292	
	Minimum		.26	
	Maximum		.87	
	Range		.61	
	Interquartile Range		.13	
	Skewness		.748	.687
	Kurtosis		1.802	1.334

T: Truness, P: Precision,
PMC: Planmeca Proface, Ein: EnScanH2, APP: EM3D scanner application
Rcheek: Right cheek unit, Lcheek: Left cheek unit,
Nose: nose unit, Perioral: Perioral unit, Chin: Mental unit
