# Supplementary material for: The accuracy of three-dimensional facial scan obtained from three different 3d scanners
Source: PLoS One. 2025 May 23;20(5):e0322358. doi: 10.1371/journal.pone.0322358 (PMC12101780; doi:10.1371/journal.pone.0322358)
Supplement: Attachment — Submitted filename: Response to Reviewers.docx [file pone.0322358.s004.docx]

**Nichakun Tangthaweesuk**
Residency Training Program in Oral and Maxillofacial Surgery
Department of Oral and Maxillofacial surgery
Faculty of Dentistry, Mahidol University
No. 6, Yothi Road, Ratchathewi District,
Bangkok 10400 THAILAND
prang.nikunn@gmail.com
+66871503113

11 February 2025

Editor-in-Chief
*PLOS ONE*
San Francisco, California, US

Dear Editor and Reviewers,

Thank you for accepting our research and pointing out the areas that require improvement. We have reviewed and revised the manuscript according to the feedback provided. The revisions have been categorized based on the journal requirements and the reviewers' recommendations as follows:

In the **Revised Manuscript with Track Changes** file, we have highlighted the modified text to facilitate **journal editors** and **reviewers** in their review process. The meaning of each highlight color is as follows:

- **Blue**: Newly added text in the manuscript.
- **Yellow**: Edited or revised text in the manuscript.
- **Red**: Deleted text from the manuscript.

These highlights ensure a **clear distinction** between additions, modifications, and removals, making the revision process more transparent and efficient.

Journal requirements:

1. We have revised the formatting of the manuscript, including the Title, Author, Affiliations, and manuscript body, to comply with PLOS ONE's style requirements. The revised content has been uploaded into “**Manuscript (Revised)”**, as attached.
2. Since we were unable to contact the original participants featured in the images intended for use, we have replaced Figures 1–3 with new photographs of other study participants. The updated image files for Figures 1, 2, and 3 have been uploaded accordingly.

Additionally, we have obtained informed consent from the new participants for the use of their 3D facial photographs, as presented in Figures 1, 2, 3, and S1, in full compliance with PLOS ONE’s policy.

The consent was provided using the PLOS open-access (CC-BY) license form to grant specific permission for the publication of these details under the terms of this license. The participants have granted their permission and signed the consent form, which is provided as evidence in the attached file titled **"Consent Form for Publication in a PLOS Journal."**Additionally, we have revised the manuscript to include the statement: **“The individual in this manuscript has given written informed consent to publish these case details.”**This amendment has been made in the **Methods** section in the attached file titled **"** **Revise Manuscript with Track Changes”** on Page [6].

3. We have revised the manuscript to ensure that the Ethics Statement appears only in the Methods section, as requested. This change has been implemented in the attached file titled **"** **Revise Manuscript with Track Changes”** on Page [36].

Reviewers' comments:

**Reviewer #1:**

We extend our sincere gratitude to Reviewer #1 for dedicating their time to thoroughly reviewing our research and offering insightful suggestions to enhance the quality of the manuscript. We have carefully reviewed and addressed the comments provided, with the revisions summarized as follows:

**• Citing references in the text of the manuscript does not meet the journal's requirements.**
: We have revised the formatting of the references to comply with the journal's requirements. All references in the original manuscript have been updated to align with the reference style outlined by the International Committee of Medical Journal Editors (ICMJE), as reflected in the attached file titled **"** **Revise Manuscript with Track Changes”** on Page [38-43].

**• Then describing “experimental instruments”, you need to indicate what iPhone/iPad model you used with EM3D Scanner app directly in corresponding section**	: In the Experimental Instruments section, we have added specific details regarding the iPhone/iPad model used in the study in conjunction with the EM3D Scanner app. The revisions are reflected in the attached file titled **"Manuscript (Revised)"** and are highlighted in blue in the file **"Revised Manuscript with Track Changes,"** on Page [8].

**• There is no data provided about the scanned surfaces in STL format: number of edges, vertices. It is important then comparing scans of different scanners.**
: Thank you for your suggestion. We have conducted additional measurements of the **scanned surfaces in STL format** using **Geomagic X Control software.** However, due to the **software's functional limitations,** it can only provide data on **poly face count** and **poly vertex count.**

We have included this information as **the average values** obtained from all three **3D scanners** used in the study. These updates have been incorporated into the **Modification, Trim, and Division of Face Models** section under **Materials and Methods.**

These revisions are included in the attached file titled **"Manuscript (Revised)"** and are highlighted in **blue** in the file **"Revised Manuscript with Track Changes,"** on Page **[11-12].**

**• I think, it is no reason to present RMS calculation formula if it was calculated by Geomagic X, not by you**
: We have removed the section on the RMS calculation formula as per the recommendations. The revisions are reflected in the attached file titled **"Manuscript (Revised)."** Additionally, the removed section is highlighted in red in the file **"Revised Manuscript with Track Changes,"** on Page [14].

**• Intraexaminer reliability was assessed only with CBCT segmentation. 3D scanning quality is more dependent on the operator – why not to assess this iterexaminer reliability?**
: Due to limited personnel, our study involved only two operators with clearly defined roles: one responsible for face model acquisition and the other for data processing and analysis using various software tools. These roles were kept separate, as the software operator is currently undergoing residency training, which requires rotations to different hospitals, making it impossible for them to participate in data collection simultaneously.

Additionally, budget constraints were a significant factor, as our study did not receive external funding. The use of the Planmeca ProFace scanner, which we accessed through the Faculty of Dentistry, Mahidol University Hospital, incurred additional costs, effectively doubling the expenses for data collection in this aspect.

Given these limitations, we were unable to implement inter-examiner reliability in our study.

**• Statistical analysis description could be more precise, clearer describing what analysis was used for what data (maybe separating by new sections).**
: In the **Statistical Analysis** section, we have revised the descriptions to make them more precise and clearer, explicitly detailing which analysis was applied to each dataset. Additionally, we have separated the descriptions into new subsections for each statistical analysis used in the study. The revisions are reflected in the attached file titled **"Manuscript (Revised)"** and are highlighted in yellow in the file **"Revised Manuscript with Track Changes,"** on Page [16].

**• There are two same tables: Table 4 on pages 18 and 19.**
: We have removed the duplicate Table 4 as recommended. The revisions are reflected in the attached file titled **"Manuscript (Revised)."** Additionally, the removed section is highlighted in red in the file **"Revised Manuscript with Track Changes,"** on Page [19].

**• What are the meanings of superscripts a,b in Tables 4 to 6?**
: We have clarified the meanings of superscripts **a, b** in **Tables 4 to 6** at the end of each table. Specifically, the superscripts **a, b i**ndicate that **post-hoc comparisons were performed using the Bonferroni test**, a statistical procedure for comparing multiple pairs of means within the data. A **p-value of <0.05** was considered statistically significant.

Our research team confirms that these superscripts are universally recognized in statistical data interpretation. **When the superscripts (a,b) are the same, it indicates no statistically significant difference between the compared pairs of data. Conversely, when the superscripts are different, it signifies a statistically significant difference between the compared pairs of data.** These updates have been included in the attached file titled **"Manuscript (Revised)"** and are highlighted in blue at the end of each table in the file **"Revised Manuscript with Track Changes,"** on Page [19,21,22].

**• Graphical representation of acquired/calculated data would be very useful – frequently it is more informative to the reader than just tables with numbers.**
: Thank you for the suggestion. We have created graphical representations of the acquired/calculated data to enhance the presentation of the results from Tables 4, 5, and 6. These graphs aim to provide a clearer and more accessible visualization for readers. The newly added figures, titled **Fig 4**, **Fig 5**, **Fig 6 and Fig 7**, correspond to the data from each table, respectively, and have been uploaded accordingly. We have revised the manuscript to include references to the newly added figures, ensuring they correspond appropriately with **Tables 4, 5, and 6**. These updates have been incorporated into the attached file titled **"Manuscript (Revised)"** and are highlighted in blue in the file **"Revised Manuscript with Track Changes,"** on Page [18-22].

**• Question about “marker stickers” – we can see on figures, what they are visible on reference 3D surfaces, but not on scanners surfaces (as you mention on page 30). From the images presented we can see that they are elevated or recessed and so they have impact on the data statistics. Could you comment on this?**
: Thank you for your valuable suggestion. We have carefully considered the potential issues related to the use of marker stickers. To address this, we conducted an additional analysis by removing the marker stickers from a subset of the reference models and re-evaluating the accuracy in terms of both trueness and precision.

The statistical analysis revealed that the accuracy results remained consistent, showing no differences between the models with and without marker stickers, as presented in the attached table.

| **Experimental instrument** | **Trueness** | |  |  | Partial Eta Squared | **Precision** | |  |  | Partial Eta Squared |
| --- | --- | --- | --- | --- | --- | --- | --- | --- | --- | --- |
|  | Mean | SD | F-test | P-value |  | Mean | SD | F-test | P-value |  |
| Planmeca ProFace |  |  |  |  |  |  |  |  |  |  |
|  | 0.83^ab^ | 0.08 | 90.169 | 0.002* | 0.984 | 0.79^ab^ | 0.10 | 19.55 | 0.019* | 0.929 |
| EinScan H2 |  |  |  |  |  |  |  |  |  |  |
|  | 0.93^a^ | 0.04 |  |  |  | 0.94^a^ | 0.05 |  |  |  |
|  |  |  |  |  |  |  |  |  |  |  |
| EM3D Scanner application |  |  |  |  |  |  |  |  |  |  |
|  | 0.66^b^ | 0.09 |  |  |  | 0.62^b^ | 0.13 |  |  |  |

We hypothesize that this outcome is due to the marker stickers occupying only a small portion of the surface area being compared, and they are present in limited regions. As a result, their presence or absence has minimal impact on the overall accuracy when performing the comparison.

**• Language of the manuscript should be clearer, more concise and with no errors – moderate English language editing is needed.**
: We have made efforts to refine the language of the manuscript, ensuring it is clearer, more concise, and free of errors. Please review the revisions. These updates have been included in the attached file titled **"Manuscript (Revised)."**

**• On page 13: “Two groups were generated, namely, the best-fit algorithm, which automatically determined the best-fit alignment by means of pre-established reference points.” – it is not clear about “two groups”.**
: The term **"two groups"** refers to the **test models obtained from 3D face scans (Planmeca ProFace, EinScan H2, and EM3D Scanner application)** and the **reference model (CBCT segmentation).**

To address the unclear phrasing, we have revised the text to provide a clearer explanation. These updates have been included in the attached file titled **"Manuscript (Revised)"** and are highlighted in yellow in the file **"Revised Manuscript with Track Changes,"** on Page [13].

**• On page 14: “Furthermore, the following percentage of overlapping surfaces within the tolerance ranges was determined by the software: <…>” – then authors present the ranges of distances for reproducibility assessment, but from the sentence you expect “percentages”.**
: We have revised the explanations for the average percentage of overlapping surfaces and the tolerance ranges, making them more distinct and clearly defined as recommended. Additionally, we have clarified their significance to enhance understanding. These updates have been included in the attached file titled **"Manuscript (Revised)"** and are highlighted in yellow in the file **"Revised Manuscript with Track Changes,"** on Page [15].

**• “grater trueness” on page 24 should be changed to “grater trueness value”. Also, please clarify on page 29 “higher trueness compared”.**
: We have revised the term "greater trueness" on page 23 to "greater trueness value" as recommended. Additionally, we have updated the Discussion section to address the comparison of devices within the Einscan lineup, as reported by Amornvit et al. (2019). Their study compared the accuracy of similar experimental tools and found that the two Einscan models used in their research demonstrated higher trueness values compared to the Planmeca ProFace and the Bellus3D Face Application on the iPhone X. These updates have been incorporated into the attached file titled **"Manuscript (Revised)"** and are highlighted accordingly in yellow in the file **"Revised Manuscript with Track Changes,"** on Page [25] and [29].

**• “The PLOS Data policy requires authors to make all data underlying the findings described in their manuscript fully available without restriction <...>”. The full data used in the manuscript is not available directly – only “upon reasonable request”.**
: We acknowledge the error and misunderstanding regarding the inclusion of the supporting file. We have corrected and updated this section accordingly. These updates have been incorporated into the attached file titled **"Manuscript (Revised)"** and are highlighted in yellow in the file **"Revised Manuscript with Track Changes,"** on Pages 31 and 43.

**Reviewer #2:**

We extend our sincere gratitude to Reviewer #2 for dedicating their time to thoroughly reviewing our research and offering insightful suggestions to enhance the quality of the manuscript. We have carefully reviewed and addressed the comments provided, with the revisions summarized as follows:

**1. Please supplement information on whether the scanning data were collected in a closed environment and whether the light source intensity was consistent during the scanning process for each device.**
: We have added information regarding the location where the 3D face scan data was collected as follows:

All 3D face scanners were used to collect data within the same closed environment, specifically in the imaging room for cone beam computed tomography (CBCT) located in the Department of Radiology, Faculty of Dentistry, Mahidol University. This room is a fully enclosed space with no windows, ensuring that no natural light enters the environment. The room is illuminated solely by LED 22W Daylight bulbs. These updates have been included in the attached file titled **"Manuscript (Revised)"** and are highlighted in blue in the file **"Revised Manuscript with Track Changes,"** on Page [9].

**2. There is an error in the description of the results section: "the Einscan H2 obtained…0.85 ± 0.8 mm" should be corrected.**
: We have corrected the numerical error in the description of the results as identified in the review. The revised statement now reads: "the EinScan H2 obtained an overall trueness value of 0.85 ± 0.28 mm." These updates have been included in the attached file titled **"Manuscript (Revised)"** and are highlighted in yellow in the **file "Revised Manuscript with Track Changes,"** on Page [18].

**3. Table 4 appears duplicate in the manuscript.**
: We have removed the duplicate Table 4 as recommended. The revisions are reflected in the attached file titled **"Manuscript (Revised)."** Additionally, the removed section is highlighted in red in the file **"Revised Manuscript with Track Changes,"** on Page [19].

**4. The experimental results of this study indicate variations in scanning accuracy across different facial regions. How can the authors demonstrate that these differences in scanning accuracy are not caused by micro-movements of facial muscles?**

: We acknowledge the impact of **micro-movements of facial muscles** on the **accuracy of 3D face scans.** To minimize this effect, we carefully designed the study to **reduce data collection time** for each participant as much as possible. All scans were conducted in a **single controlled environment,** ensuring **consistent environmental factors** across all **3D face scanners.** Participants underwent data collection in a **continuous sequence** to further minimize movement-related variations.

However, eliminating **micro-movements of facial muscles** entirely was not feasible, as the **three 3D face scanners** used in this study do not utilize **stereophotogrammetry technology**, which captures images within milliseconds.

Additionally, as our study did not record the **scanning time** for each experimental instrument, further discussion on this aspect is not possible. Nevertheless, we ensured that the **overall scanning time** for all three devices was **controlled** and **conducted by a well-trained operator,** reducing scanning delays between devices as much as possible. On average, scanning for each device was completed in **less than one minute.**

Despite the presence of **micro-movement factors,** the **reported accuracy** showed that all **test models** demonstrated **scanning accuracy across different facial regions,** with deviations remaining **less than 1.0 mm,** which is considered highly acceptable. When evaluated for **practical application**, these scanners remain a viable option, as **clinical acceptable scanning accuracy** is typically defined as **less than or equal to 1.5 mm.**

**5. Although the authors have mentioned various mesh data registration methods in the discussion, they have not clearly explained why the best-fit registration method was chosen. In fact, this registration method is not the most ideal, and the literature has mentioned registration methods based on the extraoral scan body (ESB). Please provide a comparison of these methods.**
: We have revised and provided a clearer explanation regarding the **indications for selecting the best-fit registration** method. Given that research on **mesh data registration methods based on the extraoral scan body (ESB)** remains limited, we have incorporated relevant studies to enhance clarity. Additionally, to avoid confusion with our research focus, we have removed certain **literature discussions on intraoral scanning.**

These updates have been included in the attached file titled **"Manuscript (Revised)"** and are highlighted in blue, yellow and red in the file **"Revised Manuscript with Track Changes,"** on Page **[31-32,43].**

Thank you for considering our submission. We look forward to your feedback and would be happy to address any questions or provide additional information.

Sincerely,

**Nichakun Tangthaweesuk**
Residency Training Program in Oral and Maxillofacial Surgery
Department of Oral and Maxillofacial surgery, Faculty of Dentistry, Mahidol University
