# Supplementary material for: The accuracy of three-dimensional facial scan obtained from three different 3d scanners
Source: PLoS One. 2025 May 23;20(5):e0322358. doi: 10.1371/journal.pone.0322358 (PMC12101780; doi:10.1371/journal.pone.0322358)
Supplement: Attachment — Submitted filename: Response to Reviewers (minor revision).docx [file pone.0322358.s005.docx]

**Nichakun Tangthaweesuk**
Residency Training Program in Oral and Maxillofacial Surgery
Department of Oral and Maxillofacial surgery
Faculty of Dentistry, Mahidol University
No. 6, Yothi Road, Ratchathewi District,
Bangkok 10400 THAILAND
prang.nikunn@gmail.com
+66871503113

11 February 2025

Editor-in-Chief
*PLOS ONE*
San Francisco, California, US

Dear Editor and Reviewers,

Thank you for accepting our research and pointing out the areas that require improvement. We have reviewed and revised the manuscript according to the feedback provided. The revisions have been categorized based on the journal requirements and the reviewers' recommendations as follows:

In the **Revised Manuscript with Track Changes** file, we have highlighted the modified text to facilitate **journal editors** and **reviewers** in their review process. The meaning of each highlight color is as follows:

- **Blue**: Newly added text in the manuscript.
- **Yellow**: Edited or revised text in the manuscript.
- **Red**: Deleted text from the manuscript.

These highlights ensure a **clear distinction** between additions, modifications, and removals, making the revision process more transparent and efficient.

Journal requirements:

**Please review your reference list to ensure that it is complete and correct. If you have cited papers that have been retracted, please include the rationale for doing so in the manuscript text, or remove these references and replace them with relevant current references. Any changes to the reference list should be mentioned in the rebuttal letter that accompanies your revised manuscript. If you need to cite a retracted article, indicate the article’s retracted status in the References list and also include a citation and full reference for the retraction notice.**

: We have thoroughly reviewed and verified all **references** cited in our study and confirmed that **none of them** are **retracted articles.**

Additionally, we have revised the formatting of **Reference [32]**, which cites the **International Organization for Standardization (ISO 5725-1:2023).** This reference defines the correct terminology for **accuracy (trueness and precision)** but is not in the format of a standard research article. We have adjusted it to comply with the **ICMJE reference formatting** as required by the journal.

These updates have been included in the attached file titled **"Manuscript (Minor revision)"** and are highlighted in yellow in the file **"Revised Manuscript with Track Changes (Minor revision)"** on Page **[38].**

**We note that your Data Availability Statement is currently as follows: "All relevant data are within the manuscript and its Supporting Information files." Please confirm at this time whether or not your submission contains all raw data required to replicate the results of your study. Authors must share the “minimal data set” for their submission.**

: We have added the data set relevant to the research data, which is included in the supporting file.

These updates have been included in the attached file titled **"Manuscript (Minor revision)"** and are highlighted in yellow in the file **"Revised Manuscript with Track Changes (Minor revision)"** on Page **[41].**

Reviewers' comments:

**Reviewer #1:**

We extend our sincere gratitude to Reviewer #1 for dedicating their time to thoroughly reviewing our research and offering insightful suggestions to enhance the quality of the manuscript. We have carefully reviewed and addressed the comments provided, with the revisions summarized as follows:

**• I think the English still needs editing. Also check formatting then you are citing publications, as example: "sensitivity sensors. [22,25]" -> sensitivity sensors [22,25]." - see examples of previously published articles.**
: We have thoroughly reviewed and corrected the grammar and language in the manuscript to ensure clarity and accuracy. Additionally, we have revised and improved the formatting of cited publications in accordance with your recommendations.

These updates have been included in the attached file titled **"Manuscript (Minor revision)"** and are highlighted in yellow in the file **"Revised Manuscript with Track Changes (Minor revision)"**.

Thank you for considering our submission. We look forward to your feedback and would be happy to address any questions or provide additional information.

Sincerely,

**Nichakun Tangthaweesuk**
Residency Training Program in Oral and Maxillofacial Surgery
Department of Oral and Maxillofacial surgery, Faculty of Dentistry, Mahidol University
